# Complexity in unclassified auto-inflammatory disease: a case report illustrating the potential for disease arising from the allelic burden of multiple variants

**DOI:** 10.1186/s12969-019-0374-x

**Published:** 2019-10-28

**Authors:** Lori B. Tucker, Lovro Lamot, Iwona Niemietz, Brian K. Chung, David A. Cabral, Kristin Houghton, Ross E. Petty, Kimberly A. Morishita, Gillian I. Rice, Stuart E. Turvey, William T. Gibson, Kelly L. Brown

**Affiliations:** 1 Department of Pediatrics, The University of British Columbia Faculty of Medicine, BC Children’s Hospital, 4480 Oak Street, Vancouver, BC V6H 3V4 Canada; 2 The Division of Rheumatology at British Columbia Children’s Hospital, 4480 Oak Street, Vancouver, BC V6H 3V4 Canada; 3 Department of Microbiology and Immunology, The University of British Columbia Faculty of Science, Health Sciences Mall, Vancouver, BC V6T 1Z3 Canada; 4 BC Children’s Hospital Research Institute, Rm A4-145, 950 West 28th Ave, Vancouver, BC V5Z 4H4 Canada; 5 Department of Medical Genetics, The University of British Columbia Faculty of Medicine, 4500 Oak Street, Vancouver, BC V6H 3N1 Canada; 6 Present Address: Norwegian PSC Research Center, Department of Transplantation Medicine, Division of Surgery, Inflammatory Medicine and Transplantation, Oslo University Hospital Rikshospitalet, Postboks 4950, Nydalen, N-0424 Oslo, Norway; 7 Division of Evolution and Genomic Sciences, School of Biological Sciences, Faculty of Biology Medicine and Health, University of Manchester, Manchester Academic Health Science Centre, Oxford Rd, Manchester, M13 9PL UK

**Keywords:** Autoinflammatory disease, Periodic fever syndrome, Macrophage activation syndrome, Type I interferon score, NLRP12, MEFV, Interleukin-1

## Abstract

**Background:**

Despite recent advances in the diagnosis and understanding of many autoinflammatory diseases, there are still a great number of patients with phenotypes that do not fit any clinically- and/or genetically-defined disorders.

**Case presentation:**

We describe a fourteen-year-old boy who presented at two and a half years of age with recurrent febrile episodes. Over the course of the disease, the episodes increased in frequency and severity, with new signs and symptoms continuing to appear. Most importantly, these included skin changes, splenomegaly and transaminitis. Only partial control of the disease was achieved with anti-IL-1 therapy. Extensive investigation showed generalized inflammation without immune deficiency, with increased levels of serum amyloid A and several pro-inflammatory cytokines including interferon-γ, as well as an increased type I interferon score. Exome sequence analysis identified P369S and R408Q variants in the MEFV innate immunity regulator, pyrin (*MEFV)* gene and T260 M and T320 M variants in the NLR family pyrin domain containing 12 (*NLRP12*) gene.

**Conclusion:**

Patients with unclassified and/or unexplained autoinflammatory syndromes present diagnostic and therapeutic challenges and collectively form a substantial part of every cohort of patients with autoinflammatory diseases. Therefore, it is important to acquire their full genomic profile through whole exome and/or genome sequencing and present their cases to a broader audience, to facilitate characterization of similar patients. A critical mass of well-characterized cases will lead to improved diagnosis and informed treatment.

## Background

The past 20 years have seen the identification of numerous monogenic autoinflammatory diseases with systemic and/or organ-specific inflammatory features. These features include recurrent and episodic periodic fever, serositis, arthritis, cutaneous inflammation, heightened production of IL-1β and innate immune cell activation [1]. Study of these monogenic “experiments of nature” with increasingly gene-agnostic DNA testing has extended our knowledge of the immune response, by identifying new disease-causing genes and inflammatory pathways. This process has markedly improved the timeliness and accuracy of diagnosis, and has informed treatment - especially for individuals with atypical presentations [2]. Nevertheless, among patients with clinical signs and symptoms of systemic inflammatory disease, at least 40% do not fit any of the clinically-defined diseases, and approximately 50% have no known pathogenic mutation in genes that are annotated to this family of diseases [3, 4]. Thus, there is a clear need for more detailed investigation into disease etiology, through broader interrogation of genetic variants (exome or whole genome sequencing) and complementary translational research studies aimed at improved molecular and cellular phenotyping of disease. Here we present one such case, a boy with a severe and persistent systemic autoinflammatory disorder that, despite identification of rare genetic variants and functional immunologic abnormalities, does not fit any currently-described diagnostic entity.

## Case presentation

A 4-year-old boy was referred to the Rheumatology Clinic at the British Columbia Children’s Hospital for evaluation of recurrent rash and fevers. Raised tender erythematous plaques on his limbs had been present intermittently since age 18 months, lasting for hours to weeks. Intermittent fevers to 39 °C, beginning in the evening, and lasting for days to weeks, began at 2.5 years of age, unassociated with the skin lesions, and without identifiable precipitants. Symptoms associated with the fevers included lethargy, arthralgia, myalgia, headache, cough, vomiting and loose stool.

The parents are unrelated, and of East Indian Kenyan ancestry. No relative has symptoms similar to those of the patient. The boy was the product of a term pregnancy complicated by maternal antiphospholipid syndrome (resulting in three fetal losses prior to 6 weeks’ gestation)*,* Graves’ disease (for which his mother had received previous radiation), and maternal thyroxine during the pregnancy. His father and two siblings are healthy. As part of the investigations of recurrent fetal loss, karyotype analysis indicated that the father carried an apparently-balanced reciprocal translocation involving the long arms of chromosome 5 and 14 with karyotype 46,XY, t (5, 14)(q11.2;q32.1).

Initial laboratory investigations of the patient at age 4 years revealed elevated ESR, mild anemia, and low leukocyte and platelet count. Because of concern about continuing symptoms, and the presence of an autoinflammatory syndrome, extensive inital investigations were performed with normal or negative results: quantitation of C-reactive protein (CRP), liver enzymes, ferritin, blood urea nitrogen (BUN), creatinine, urinalysis, immunoglobulins (including IgD during a febrile episode), antinuclear antibodies (ANA), anti-neutrophil cytoplasmic antibody (ANCA), anti-cardiolipin antibody (aCL), von Willebrand factor (vWF) antigen, complement (C3 and C4), tissue transglutaminase (tTG), alpha-1 antitrypsin, TB skin test and chest x-ray.

Genetic screening for Familial Mediterranean Fever (FMF) showed compound heterozygous variants of uncertain significance in exon 3 of the *MEFV* gene, p.P369S and p.R408Q. At the time of this writing, both variants have conflicting interpretations in ClinVar (www.ncbi.nlm.nih.gov/clinvar/). Subsequent analysis of the parents determined that the father was also heterozygous for both MEFV variants while the mother was negative, which led to the conclusion that the two *MEFV* variants are in *cis* position (on the same allele, thereby constituting a haplotype). Prior to genetic investigation of FMF, the patient was provisionally treated with colchicine (0.3 mg BID) and minor improvement in the recurrence, but not severity, of fever episodes was observed. Several attempts to discontinue colchicine treatment resulted in increased frequency of fever episodes, so colchicine was continued.

At 5 years of age, the skin rash became more extensive, involving both lower extremities, and had the appearance of erythema nodosum. He also developed moderate hepatomegaly with mild elevation of liver enzymes (AST range 78–158 U/L, ALT range 59–251 U/L) and elevated LDH (range 938–1616 U/L), which continued to be elevated for most of the time during the course of the disease (Table 1). He had persistent anemia and worsening pancytopenia, with negative testing for infectious diseases, inflammatory bowel disease, autoimmune hepatitis, and metabolic disease. Immunodeficiency screening, including mitogen testing, T and B cell panel and immunoglobulin levels were reported as normal or negative. Bone marrow examination showed lack of iron stores, but was otherwise unremarkable. Histopathology of a liver biopsy showed mild to moderate lobular hepatitis with hemophagocytic lymphohistiocytosis (HLH). Laboratory evaluations for HLH, including NK cell function, perforin/granzyme B percentages and soluble IL-2 receptor (sIL-2R) levels were normal, while the screening showed no mutations in MUNC13–4, PRF1, STX11 or RAB27A genes.

**Table 1:** Frequently abnormal clinical laboratory values over the course of disease^a^

	*Patient median*	*Patient range*	*Normal range*
A. Complete Blood Cell count (CBC) and liver function values			
Hb (g/L)	114	96–126	125–165
WBC (× 10^9^/L)	3	1.7–10.9	3.9–10.2
Platelets (× 10^9^/L)	172	151–231	180–440
ALT (U/L)	83	25–650	0–50
AST (U/L)	97.5	66–178	0–36
LDH (U/L)	1151	133–1754	120–300
B. Inflammatory markers			
CRP (mg/L)	5	0–45	0–5
ESR (mm)	14	4–42	0–15
Ferritin (ug/L)	70	29–465	31–177
SAA (ng/mL)	11,765	1752 – 193,855	1000–5000

^**a**^Median and range calculated from test results obtained approximately three times a year over 10 years (age 5–14 yrs). Normal ranges are age-specific values from the BC Children’s Hospital Clinical Laboratory. Changes in parameters did not correlate with alterations in clinical state and neither correlated with treatment modalities, except for a decrease in SAA upon treatment with canakinumab (Fig. 1)

Further testing was undertaken to pursue the possibility of an autoinflammatory syndrome. Sequencing of the *TNFRSF1A* and *MVK* genes revealed no variants associated with Tumor Necrosis Factor Receptor – Associated Periodic Syndrome (TRAPS) or Hyper IgD Syndrome (HIDS), respectively. Measurement of serum and cerebrospinal fluid cytokines (Mesoscale Human Biomarker 40-plex) during a flare of disease showed elevated concentrations of several pro-inflammatory cytokines, most notably, interferon (IFN)-γ (Table 2). A type 1 IFN score [5] based on the expression of 6 genes (IF127, IF144L, IFIT1, ISG15, RSAD2, SIGLEC1), was elevated (4.531) suggesting heightened activity of IFN-α and/or IFN-β. Research-based whole exome sequencing showed rare variants in the following genes:

- *NLRP12*: two heterozygous variants of unknown significance in exon 3 (p.T260 M inherited from mother and p.T320 M inherited from father) and one heterozygous variant classified as likely benign in exon 1 (p.G39 V inherited from father);
- *CEP164*: two variants classified as likely benign (homozygous p.P495R and heterozygous p.R1122C inherited from father);
- *COL6A3*: one homozygous variant of unknown significance (p.R1990W);
- *TREML1*: one previously undescribed homozygous variant causing in-frame deletion.

**Table 2:** Cytokine concentration in patient serum and cerebrospinal fluid

Cytokine	SERUM (pg/mL)		CSF (pg/mL)	
	Patient	Controls (mean ± SD)	Patient	Controls (mean ± SD)
bFGF	**2.28**	42.39 ± 32.66	**0.37**	0.12 ± 0.04
CRP (S: × 10^6^; CSF: × 10^3^)	1.62	1.42 ± 1.52	28.42	106.67 ± 173.26
Eotaxin	50.88	59.43 ± 29.41	**19.99**	3.42 ± 3.47
Eotaxin-3	0.95	20.77 ± 17.00	ND	ND
GM-CSF	**2.00**	0.44 ± 0.37	0.23	0.33 ± 012
ICAM-1 (S: ×10^3^; CSF: × 10^3^)	**749.69**	568.43 ± 82.32	**29.03**	11.95 ± 4.21
IFN-γ (S: × 10^3^)	**2.58**	0.003 ± 0.001	**12.39**	0.64 ± 0.37
IL-1β	ND	ND	ND	ND
IL-1α	0.02	0.08 ± 0.09	0.43	0.44 ± 0.56
IL-2	ND	0.35 ± 0.17	**0.02**	0.01 ± ND^a^
IL-4	0.06	0.03 ± 0.02	ND	0.03 ± ND^a^
IL-5	ND	2.58 ± ND^a^	**0.62**	0.33 ± 0.04
IL-6	**1.96**	0.29 ± 0.35	**1.67**	0.46 ± 0.14
IL-7	21.56	15.43 ± 7.24	2.21	2.64 ± 0.85
IL-8	9.76	5.35 ± 2.94	**43.93**	12.75 ± 10.52
IL-10	**1.29**	0.33 ± 0.03	0.04	ND
IL-12/IL-23p40	**616.99**	285.75 ± 70.30	**11.21**	3.26 ± 0.82
IL-12p70	0.13	0.04 ± ND^a^	ND	0.09 ± 0.05
IL-13	**0.80**	0.32 ± 0.12	ND	0.55 ± ND^a^
IL-15	**3.17**	1.78 ± 0.46	**5.11**	1.94 ± 0.45
IL-16	239.07	203.49 ± 73.13	**6.67**	2.92 ± 1.55
IL-17A	6.83	3.30 ± 2.74	ND	0.32 ± 0.44
IP-10 (S: ×10^3^; CSF: ×10^3^)	**169.40**	0.45 ± 0.16	**2.80**	0.03 ± 0.02
MCP-1	**338.23**	195.79 ± 9.00	557.74	368.26 ± 80.38
MCP-4	**21.40**	70.85 ± 7.68	6.40	3.73 ± 2.13
MDC (S: ×10^3^)	**1.07**	1.93 ± 0.09	**76.25**	2.49 ± 0.25
MIP-1α	**28.33**	9.10 ± 2.19	**18.95**	3.07 ± 1.67
MIP-1β	179.78	85.20 ± 74.99	11.32	6.48 ± 4.48
PIGF	**48.42**	24.00 ± 7.56	**15.29**	17.79 ± 0.51
SAA (S: ×10^6^; CSF: ×10^3^)	**16.24**	22.28 ± 31.34	**31.27**	214.20 ± 368.14
sFIt-1	**84.64**	90.11 ± 1.99	**35.51**	12.93 ± 7.90
TARC	147.94	370.04 ± 179.10	**3.37**	0.38 ± 0.10
Tie-2 (S: ×10^3^)	**5.75**	4.19 ± 0.99	**17.39**	9.71 ± 2.72
TNF-α	**7.98**	3.50 ± 0.96	**0.22**	0.04 ± 0.00
TNF-β	**2.43**	0.56 ± 0.51	ND	0.06 ± 0.00
VCAM-1 (S: ×10^6^; CSF: ×10^3^)	**1.31**	0.70 ± 0.05	**67.62**	19.20 ± 6.70
VEGF	**362.13**	289.56 ± 33.77	3.22	4.38 ± 1.50
VEGF	**770.44**	573.56 ± 40.83	**1.77**	3.12 ± 0.69
VEGF-C	226.30	358.58 ± 95.79	ND	8.52 ± ND^a^
VEGF-D	**304.25**	350.99 ± 119.84	**21.61**	3.04 ± 2.40

Cytokine concentrations in serum (S) and cerebrospinal fluid (CSF) from the patient and children (*n* = 3 for serum, *n* = 3 for CSF) admitted to BC Children’s Hospital for non-inflammatory conditions. Patient values that were 2 SD below or above control mean values are shown in bold

*ND* no data; values below the lower range of detection: pg/ml, Eotaxin 3; 3.26, IL-1β; 0.04, IL-2; 0.09, IL-4; 0.02, IL-5; 0.22, IL-10; 0.03, IL-12p70; 0.11, IL-13; 0.24, IL-17A; 0.74, TNF-β; 0.05, VEGF-C; 11.1

^a^SD was not calculated because values were below the level of detection for at least one individual

Sanger sequencing of the *NLRP12* gene in the patient, parents and siblings, showed that the variants did not confidently co-segregate with disease status. Variants in the *MEFV* gene and the *NLRP12* gene were subsequently confirmed by clinical-based whole exome sequencing (Table 3).

**Table 3:** Gene variants^a^ associated with patient’s clinical phenotype

Gene	Disease	Mode of inheritance	Variant	Coding DNA	Zygosity	Inherited from	^b^Classification
*NLRP12*	NLRP12-related disorder	Autosomal dominant / recessive	T260 M	c.779 C > T	Heterozygous	Mother	Variant of Unknown Significance
*NLRP12*	NLRP12-related disorder	Autosomal dominant / recessive	T320 M	c.959 C > T	Heterozygous	Father	Variant of Unknown Significance
*NLRP12*	NLRP12-related disorder	Autosomal dominant / recessive	G39V	c.116 G > T	Heterozygous	Father	Likely benign
*MEFV*	FMF	Autosomal dominant / recessive	P369S	c.1105C > T	Heterozygous	Father	Variant of Unknown Significance
*MEFV*	FMF	Autosomal dominant / recessive	R408Q	c.1223G > A	Heterozygous	Father	Variant of Unknown Significance

^a^variants were discovered by WES and confirmed by Sanger sequencing

^b^interpretations from Clinvar at the time of writing

**Fig. 1:**
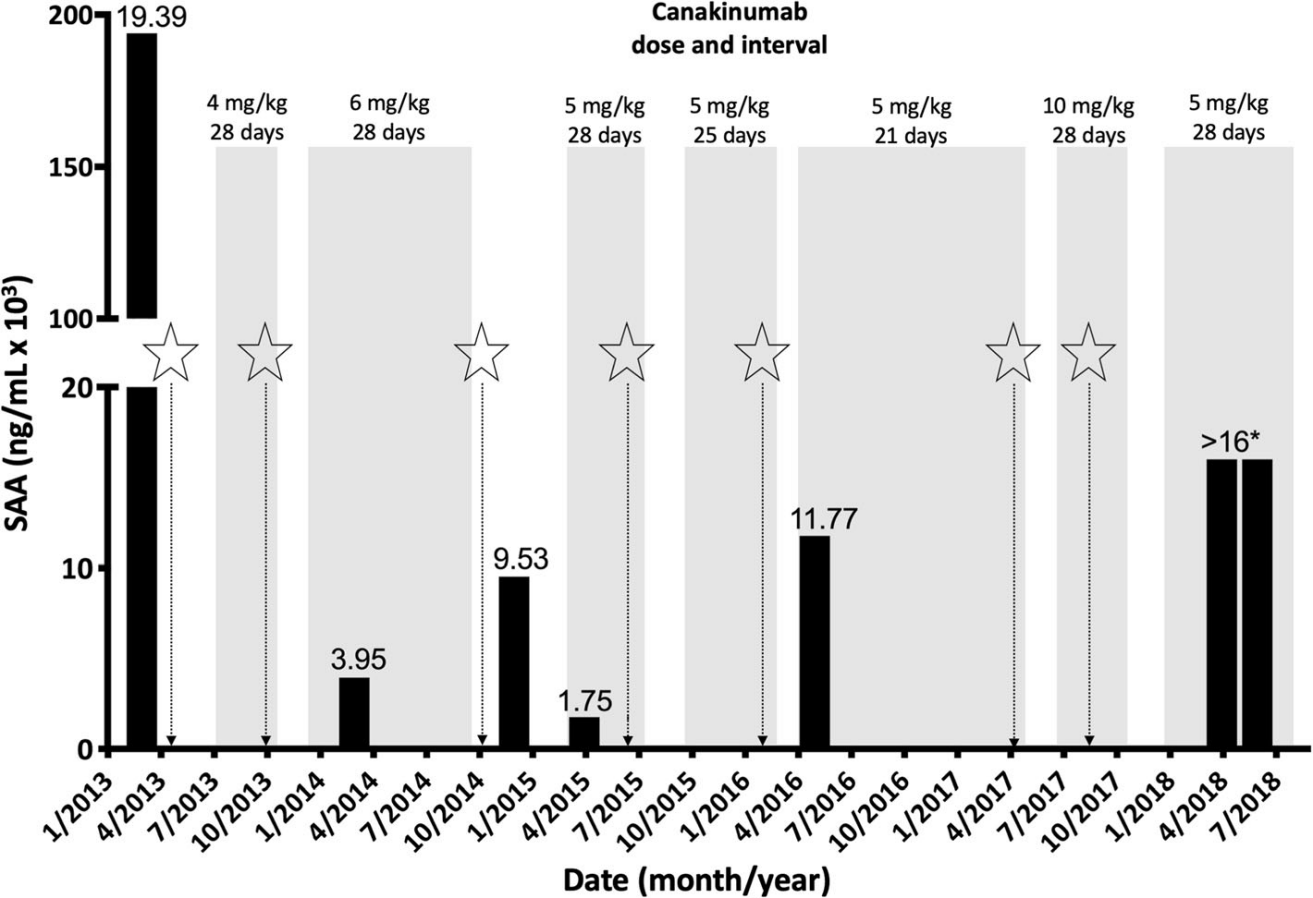
Timeline of canakinumab treatment, serum amyloid (SAA) concentration, and major disease flares. Bars show SAA concentrations (y-axis, ng/ml × 10^3^, performed at Dynacare Laboratories, Canada; * exceeded upper limit of detection). Grey shading shows the start and end of periods (x-axis, month/year) of canakinumab treatment with doses (mg/kg) and duration (days) annotated above. Stars represent major disease flares lasting for more than 5 days and characterized by increased temperature, skin changes, abdominal pain and/or headaches

At age 6 years, a diagnosis of periodic fever syndrome, unclassified, with associated macrophage activation syndrome (MAS) features was given. Treatment with oral prednisone (0.5–1 mg/kg/day) resulted in slight improvement, but the patient continued to have episodes of illness. His growth during this period was poor, he missed substantial amounts of school and he was unable to participate in sports and other extracurricular activities. He was started on Anakinra at 2 mg/kg/day with minimal improvement, but with a dose increase to 4 mg/kg/day, his fever episodes became shorter, new lesions of the erythema nodosum-like rash on the lower extremity were present on a less frequent basis, and he had less need for supplemental corticosteroids. Despite these improvements, he continued to have febrile episodes every 1–2 months, lasting 1–2 weeks, with accompanying painful skin nodules, hepatosplenomegaly, pancytopenia, and raised liver enzymes. A dose increase of Anakinra to 8 mg/kg/day did not provide any incremental symptomatic improvement and the drug (but not prednisone) was discontinued because of severe urticarial skin lesions. Following anakinra discontinuation, serum amyloid A (SAA) levels were extremely high (193,855 ng/mL, normal range 1000–5000 ng/mL) (Fig. 1).

At age 8.5 years, in the face of continuing disease, canakinumab at 4 mg/kg every 4 weeks was started and the patient had rapid and complete resolution of fever, musculoskeletal pain, and fatigue; this enabled tapering of prednisone and correlated with improved laboratory tests, including a substantial drop in SAA (to 5000 ng/mL). Nevertheless, he continued to have occurence of erythema nodosum-like lesions primarily on the lower extremity, and he had several episodes of fever requiring intermittent increases in prednisone dose. After 1 year, the canakinumab was discontinued for a period of time due to funding coverage issues. This led to a significant flare that, regardless of daily high dose prednisone, subsided only after canakinumab was restarted. During the following year he started to have more frequent fever episodes occurring just prior to the canakinumab dose. He had episodes of severe abdominal pain with splenomegaly, and he suffered from frequent headaches, often independent of fever. Ultrasound and fibroscan were negative for liver fibrosis, cirrhosis and portal hypertension. An extensive neurological workup that included brain imaging (MRI/MRA/MRV), ophthalmology assessment and lumbar puncture, was normal. Eventually, the canakinumab dose interval was reduced from 28 days to 21 days.

After 4 years of canakinumab treatment, the patient continued to have episodes of fever every 1–2 months and frequent erythema-nodosum lesions. He had a trial of increased canakinumab dose from 150 mg (5 mg/kg) every 21 days to 300 mg (10 mg/kg) every 4 weeks. After initial improvement on the higher dose, with fever occurring only every 3–4 months and lasting only for 1 day, parents noted that the patient had an increasing frequency of disease flares, occurring monthly. Canakinumab was decreased to 150 mg every 4 weeks, with regular daily colchicine and continual low dose prednisone that is increased with febrile flares. This combination of medications has resulted in a relatively stable clinical situation without any major febrile illness episodes. The patient has sustained modest linear growth, and has maintained school attendance and participation.

## Discussion

The patient we describe is typical of the many atypical and unexplained patients seen in pediatric rheumatology clinics. His predominant clinical symptoms - periodic fever, recurrent erythema nodosum-like lesions, persistent splenomegaly and transaminitis were suggestive of several autoinflammatory diseases, but not one specifically. Hemophagocytic lymphohistiocytosis syndrome/macrophage activation syndrome (HLH/MAS) was thought to be present based on the patient’s clinical findings of fever, splenomegaly, as well as laboratory findings of pancytopenia and liver biopsy showing tissue hemophagocytosis. However, the entirety of the patient’s disease course and lab findings suggest that the HLH/MAS has been a secondary phenomenon [6]. Although he has been ill for most of his life and had extensive diagnostic workup and genetic testing, he does not yet have a definitive diagnosis.

Exome sequencing identified numerous variants of unknown significance in genes known to be associated with periodic fever syndromes, namely two important autoinflammatory genes, *MEFV* and *NLRP12*. P369S and R408Q variants are found in exon 3 of the *MEFV* gene and appear to be in linkage disequilibrium [7]. When in *cis* position, they are often associated with a highly variable phenotype, and infrequently with typical FMF symptoms [7, 8]. Additionally, published literature reports that these variants both in *cis* and/or *trans* position are found in patients with PFAPA, Henoch-Schönlein purpura, inflammatory myopathies, protracted febrile myalgia syndrome and Behcet’s disease [9–12]. Related to this was the report of a patient heterozygous for P369S and R408Q variants with systemic lupus erythematosus-associated MAS [13]. Patients with these variants and atypical FMF presentation can later develop AA amyloidosis due to the deposition of SAA, even without mutations in exon 10, necessitating consideration of colchicine in symptomatic carriers [7, 14]. Finally, these variants are also associated with later presentation of FMF [15]. Together these reports indicate the importance of P369S and R408Q variants in diseases with an autoinflammatory component, although in silico modeling suggests that these variants do not result in significant modifications of pyrin’s function [7, 16]. The T260 M and T320 M variants in the *NLRP12* gene, inherited from mother and father in our patient, respectively, have not been annotated to phenotypes in the literature, to our knowledge. T260 M is represented in the ExAC and gnomAD databases in 81 and 153 individuals, respectively, whereas T320 M is represented in these same databases in 4 and 11 individuals, respectively [17].

NLRP12-associated hereditary periodic fever syndrome is a rare autoinflammatory syndrome characterized by episodic and recurrent periods of fever combined with various systemic manifestations such as myalgia, arthralgia, joint swelling, urticaria, headache and skin rash. Common trigger of these episodes is cold, which was not evident in our patient.

This patient’s clinical symptoms and course were also not completely consistent with FMF attacks, which are usually brief episodes of fever accompanied by peritonitis, pleurisy, arthritis or skin rash. Our patient’s disease episodes were prolonged, and the skin findings not consistent with an FMF rash. The importance of FMF heterozygosity in clinical situations has been controversial. A population genotype study of four Mediterranean groups done by Jeru et al. demonstrated that individuals with a heterozygous MEFV mutation were at increased risk of developing FMF with a relative risk of 6.3–8.1 compared to non-carriers [18].

Although none of this patient’s rare variants met criteria to be regarded as pathogenic, we speculate that in aggregate they might confer an “allelic burden,” such that their combination could create an autoinflammatory milieu consisting of increased pro-inflammatory cytokines, SAA and both type I and type II IFN. The operation of more than one underlying mechanism could also help to explain why the response to different treatment modalities, including complete blockade of IL-1 (as suggested by cytokine analysis; Table 2), was variable – with incomplete control of inflammation and persistence of some milder symptoms. The issue of allelic burden is well-described and well-accepted as it relates to Polygenic Risk Scores (PRS) derived from Genome-Wide Association Studies (GWAS). However, PRS rely on the identification of common variants that individually confer a small odds ratio for disease, whereas the additive effects of rare alleles conferring moderate risk for disease have proven more difficult to characterize. Additional complexity ensues when more than one rare allele is present in the same person, as the overlapping and/or synergistic effects of genetic “hits” in more than one inflammatory pathway are difficult to deconvolute without detailed assays that interrogate mechanism at multiple levels. Furthermore, the intrinsic variability of autoinflammatory phenotypes may make them impossible to predict from genotype alone.

## Conclusion

In conclusion, we would like to emphasize the challenges faced by patients with unclassified / unexplained autoinflammatory syndromes that may persist even after a thorough workup with contemporary technologies. Although each case is unique and extremely rare, together this group of patients represents a substantial proportion of every autoinflammatory cohort. Therefore, we strongly encourage the development of bioinformatic mechanisms and clinical registries to aggregate similar patients together creating a common ground for further characterization of new autoinflammatory entities [19].

## Data Availability

Not applicable.

## Abbreviations

aCL: Anti-cardiolipin antibody
ALT: Alanine aminotransferase
ANA: Antinuclear antibody
ANCA: Anti-neutrophil cytoplasmic antibody
AST: Aspartate aminotransferase
bFGF: Basic fibroblast growth factor
BUN: Blood urea nitrogen
CRP: C-reactive protein
FMF: Familial Mediterranean fever
GGT: Gamma-glutamyl transferase
GM-CSF: Granulocyte-macrophage colony-stimulating factor
HIDS: Hyper IgD syndrome
HLH: Hemophagocytic lymphohistiocytosis
ICAM1: Intercellular Adhesion Molecule 1
IFNγ: Interferon gamma
IL-1: Interleukin 1
IL-10: Interleukin 10
IL-13: Interleukin 13
IL-15: Interleukin 15
IL-16: Interleukin 16
IL-17A: Interleukin 17A
IL-1α: Interleukin 1 alpha
IL-1β: Interleukin 1 beta
IL-4: Interleukin 4
IL-5: Interleukin 5
IL-7: Interleukin 7
IL-8: Interleukin 8
IP-10: Interferon gamma-induced protein 10
LDH: Lactate dehydrogenase
MAS: Macrophage activation syndrome
MCP-1: Monocyte chemoattractant protein 1
MEFV: MEFV innate immunity regulator, pyrin
MIP-1α: Macrophage inflammatory protein 1 alpha
MIP-1β: Macrophage inflammatory protein 1 beta
MRA: Magnetic resonance angiography
MRI: Magnetic resonance imaging
MRV: Magnetic resonance venography
NLRP12: Nucleotide-binding domain and leucine-rich repeat family pyrin domain containing 12
PIGF: Phosphatidylinositol-glycan biosynthesis class F protein
SAA: Serum amyloid A
sFIT-1: Soluble fms-like tyrosine kinase 1
sIL-2R: Soluble interleukin 2 receptor
TARC: Thymus and activation-regulated chemokine
TB: Tuberculosis
Tie-2: Angiopoietin receptor
TNFα: Tumor necrosis factor alpha
TNFβ: Tumor necrosis factor beta
TRAPS: Tumor necrosis factor receptor – associated periodic syndrome
tTG: Tissue transglutaminase
VCAM-1: Vascular cell adhesion molecule 1
VEGF: Vascular endothelial growth factor
VEGF-C: Vascular Endothelial Growth Factor C
vWF: Von Wilebrand factor antigen

## References

[1] Masters SL, Simon A, Aksentijevich I, Kastner DL (2009). Horror autoinflammaticus: the molecular pathophysiology of autoinflammatory disease (*). Annu Rev Immunol.

[2] Marcuzzi A, Piscianz E, Kleiner G, Tommasini A, Severini GM, Monasta L (2013). Clinical genetic testing of periodic fever syndromes. Biomed Res Int.

[3] Russo RA, Brogan PA (2014). Monogenic autoinflammatory diseases. Rheumatology (Oxford).

[4] Lachmann HJ (2017). Periodic fever syndromes. Best Pract Res Clin Rheumatol.

[5] Rice GI, Forte GM, Szynkiewicz M, Chase DS, Aeby A, Abdel-Hamid MS, et al. Assessment of interferon-related Rice GI, Forte GM, Szynkiewicz M, Chase DS, Aeby A, Abdel-Hamid MS, et al. Assessment of interferon-related biomarkers in Aicardi-Goutieres syndrome associated with mutations in TREX1, RNASEH2A, RNASEH2B, RNASEH2C, SAMHD1, and ADAR: a case-control study. Lancet Neurol. 2013;12(12):1159-69. 10.1016/S1474-4422(13)70258-8 PMC4349523 24183309

[6] Sen ES, Steward CG, Ramanan AV (2017). Diagnosing haemophagocytic syndrome. Arch Dis Child.

[7] Ryan JG, Masters SL, Booty MG, Habal N, Alexander JD, Barham BK (2010). Clinical features and functional significance of the P369S/R408Q variant in pyrin, the familial Mediterranean fever protein. Ann Rheum Dis.

[8] Westwell-Roper C, Niemietz I, Tucker L, Brown K. Periodic fever syndromes: beyond the single gene paradigm. Pediatr Rheumatol Online J. (Accepted for publication). 10.1186/s12969-019-0324-7 PMC6515597 31088470

[9] Yamagami K, Nakamura T, Nakamura R, Hanioka Y, Seki K, Chiba H (2017). Familial Mediterranean fever with P369S/R408Q exon3 variant in pyrin presenting as symptoms of PFAPA. Mod Rheumatol.

[10] Fujikawa K, Migita K, Shigemitsu Y, Umeda M, Nonaka F, Tamai M (2014). MEFV gene polymorphisms and TNFRSF1A mutation in patients with inflammatory myopathy with abundant macrophages. Clin Exp Immunol.

[11] Fujikawa K, Migita K, Tsukada T, Kawakami A, Eguchi K (2014). Protracted febrile myalgia syndrome in a Japanese patient with fasciitis detected on MRI. Intern Med.

[12] Fujikawa K, Migita K, Nagasato A, Tsukada T, Kawakami A, Eguchi K (2014). Mediterranean fever (MEFV) variant P369S/R408Q in a patient with entero-Behcet's disease who successfully responded to treatment with colchicine. Intern Med.

[13] Shimizu M, Yokoyama T, Tokuhisa Y, Ishikawa S, Sakakibara Y, Ueno K (2013). Distinct cytokine profile in juvenile systemic lupus erythematosus-associated macrophage activation syndrome. Clin Immunol.

[14] Yabuuchi J, Hayami N, Hoshino J, Sumida K, Suwabe T, Ueno T (2017). AA amyloidosis and atypical familial Mediterranean fever with exon 2 and 3 mutations. Case Rep Nephrol Dial.

[15] Hannan LM, Ward J, Ebringer R, McDonald CF (2012). Late presentation of familial Mediterranean fever associated with P369S/R408Q variant in the MEFV gene. Intern Med J.

[16] Yu JW, Fernandes-Alnemri T, Datta P, Wu J, Juliana C, Solorzano L (2007). Pyrin activates the ASC pyroptosome in response to engagement by autoinflammatory PSTPIP1 mutants. Mol Cell.

[17] Lek M, Karczewski KJ, Minikel EV, Samocha KE, Banks E, Fennell T (2016). Analysis of protein-coding genetic variation in 60,706 humans. Nature.

[18] Jeru I, Hentgen V, Cochet E, Duquesnoy P, Le Borgne G, Grimprel E (2013). The risk of familial Mediterranean fever in MEFV heterozygotes: a statistical approach. PLoS One.

[19] Philippakis AA, Azzariti DR, Beltran S, Brookes AJ, Brownstein CA, Brudno M (2015). The matchmaker exchange: a platform for rare disease gene discovery. Hum Mutat.

